# Ablation of the deubiquitinating enzyme cylindromatosis (CYLD) augments STAT1-mediated M1 macrophage polarization and fosters *Staphylococcus aureus* control

**DOI:** 10.3389/fimmu.2025.1507989

**Published:** 2025-01-28

**Authors:** Christina Schmidt, Kunjan Harit, Stephan Traidl, Michael Naumann, Thomas Werfel, Lennart M. Roesner, Gopala Nishanth, Dirk Schlüter

**Affiliations:** ^1^ Institute of Medical Microbiology and Hospital Epidemiology, Hannover Medical School, Hannover, Germany; ^2^ Department of Dermatology and Allergy, Hannover Medical School, Hannover, Germany; ^3^ Institute of Experimental Internal Medicine, Otto-von-Guericke-University Magdeburg, Magdeburg, Germany; ^4^ Cluster of Excellence RESIST (EXC 2155), Hannover Medical School, Hannover, Germany

**Keywords:** *Staphylococcus aureus*, macrophage, CYLD, ubiquitin, atopic dermatitis, STAT1, NF-κB

## Abstract

In atopic dermatitis (AD), lesional skin is frequently colonized by *Staphylococcus aureus*, which promotes clinical symptoms of the disease. The inflammatory milieu in the skin is characterized by a Th2 response, including M2 macrophages, which cannot eradicate *S. aureus*. Therefore, repolarization of macrophages toward the M1 phenotype may foster control of *S. aureus*. Our data show that the deubiquitinating enzyme cylindromatosis (*CYLD*) is strongly expressed in macrophages of AD patients and prevents the clearance of *S. aureus*. Mechanistically, *CYLD* impaired M1 macrophage polarization by K63-specific deubiquitination of STAT1 and activation of the NF-κB pathway via its interaction with TRAF6, NEMO, and RIPK2. Inhibition of STAT1 and NF-κB, independently, abolished the differences between *S. aureus*-infected *CYLD*-deficient and *CYLD*-competent M1 macrophages. Infection of *Cyld*-deficient and wild-type mice with *S. aureus* confirmed the protective CYLD function. Collectively, our study shows that *CYLD* impairs the control of *S. aureus* in macrophages of AD patients, identifying CYLD as a potential therapeutic target.

## Introduction

Eczematous skin lesions of patients with atopic dermatitis (AD) are frequently colonized with *Staphylococcus aureus*, and the extent of colonization increases in more severe lesions with hyperinflammation (1–5). These staphylococci contribute to disease symptoms, in particular itching and pain, by the secretion of toxins and proteases, which directly stimulate sensory nerves in the skin (6–8). In addition, these staphylococci may cause local and, in some cases, systemic infections in AD patients. The importance of the role of *S. aureus* in AD is supported by the fact that treatment of skin infection with antiseptics or antibiotics can improve the symptoms of AD (9).

Upon infection, control of *S. aureus* critically depends on protective innate immune responses. Neutrophils can phagocytose and kill the bacteria and also immobilize *S. aureus* through NETosis, i.e., capturing the bacteria in a dense network of DNA released by the neutrophils (10, 11). In addition, both tissue-resident and monocyte-derived macrophages contribute to the control and elimination of *S. aureus* (12). Macrophages can rapidly phagocytose *S. aureus* and activate the NF-κB pathway, which contributes to the production of anti-bacterial reactive oxygen species (ROS) and nitric oxide (13). Activation of macrophages by interferon (IFN)-γ synergizes with TLR2-mediated NF-κB activation in NO production and further enhances control of *S. aureus* in macrophages. These “M1-like” macrophages also contribute to the control of local *S. aureus* skin infection and contribute to the prevention of systemic bacterial dissemination (14). In contrast to M1 macrophages, M2-polarized macrophages have impaired anti-staphylococcal activity and capacity to control *S. aureus* infections (12). *S. aureus* can subvert these protective intracellular mechanisms to replicate and persist intracellularly in macrophages and other cell types including keratinocytes (13, 15–18).

The lesional skin of AD patients is characterized by a type 2 inflammatory milieu composed of macrophages, monocytes, B cells, CD4^+^, and CD8^+^ T cells (19, 20). This type 2 inflammatory environment may facilitate the persistence of extracellular *S. aureus* and also intracellularly in keratinocytes (16) and macrophages (17, 18). In AD patients, the dysregulation of the immune system also extends beyond the skin and includes altered systemic innate and adaptive immune profiles, leading to comorbidities including infections (21–23).

Immune responses are critically regulated by post-translational modifications including ubiquitination. Ubiquitination is a highly dynamic process exerted by a cascade of ubiquitin-activating E1, ubiquitin-conjugating E2, and ubiquitin E3 ligases, which can attach the ubiquitin protein to a lysine residue of a substrate protein, and by deubiquitinating enzymes (DUBs), which can cleave ubiquitin from the substrates. Ubiquitin can be attached as monomers or as ubiquitin chains to the substrates. In polyubiquitination, ubiquitin molecules are linked through any one of the internal seven lysines (K6, K11, K27, K29, K33, K48, and K63) or the N-terminal methionine residue (M1). The type of ubiquitin linkage decides on the function and fate of the substrate and can lead to proteasomal degradation by K48- and branched K11/K48-linked ubiquitin chains (24, 25) or to K63-linked ubiquitin modification of protein function including regulation of signal transduction (26).

Ubiquitination is reversible and counteracted by DUBs. The DUB cylindromatosis (CYLD) can cleave K63- and M1-linked polyubiquitin chains from substrates and regulates a broad range of key cellular processes including inflammatory responses, cell death pathways, autophagy, DNA damage, and cell proliferation (27, 28). In immune signaling, CYLD negatively regulates NF-κB by deubiquitinating TRAF2, TRAF6, TAK1, and NEMO (29–32). With respect to bacterial infections, this suppressive effect of CYLD on NF-κB prevented immunopathology in *Haemophilus influenzae* and *Streptococcus pneumoniae* infections (33, 34) but protective immune responses in *Escherichia coli* pneumonia and systemic listeriosis (35, 36). In addition, CYLD deubiquitinates STAT3 and RIPK2, thereby impairing protective IL-6 and NOD2-dependent protective immunity in listeriosis (37, 38).

Inactivating mutations of *CYLD* underlie the CYLD cutaneous syndrome, a disease characterized by the development of benign skin tumors of the hair follicles (39) including multiple familial trichoepithelioma, the Brooke–Spiegler syndrome, and familial cylindromatosis. In addition, somatic *CYLD* mutations have been linked to the suppression of sporadic cancers, non-alcoholic steatohepatitis (40), arterial hypertension (41), and gain-of-function mutations to neurodegenerative disorders (42) (reviewed in Marin-Rubio et al., 2023 (28)).

Although *CYLD* is strongly expressed in healthy skin and macrophages (28, 43), its expression and function in AD and *S. aureus* infections are unknown. To address these open questions, we analyzed the function of *CYLD* in AD patients. Our data show that CYLD is strongly expressed in the dermal macrophages and monocyte-derived macrophages (MDMs) of AD patients and that *CYLD* impairs STAT1- and NF-κB-dependent control of *S. aureus* in macrophages.

## Materials and methods

### Ethics statement

All animal experiments were in compliance with the German animal protection law in a protocol approved by the Landesverwaltungsamt Sachsen-Anhalt (file number: 203.h-42502-2-901, University of Magdeburg). The ethics committee of Hannover Medical School (MHH) approved the parts of the study involving patients (No. 10499-BO-K-2022).

### Animals

Age- and sex-matched animals were used for the experiments. C57BL/6 wild-type (WT) mice were obtained from Janvier (Le Genest Saint Isle, France), and C57BL/6 *Cyld* ^−/−^ mice were kindly provided by Dr. Ramin Massoumi (Department of Laboratory Medicine, Malmö, Sweden) (44). All animals were kept under specific pathogen-free (SPF) conditions in an isolation facility at the Otto-von-Guericke University Magdeburg (Magdeburg, Germany).

### Patients

Patients with AD were recruited at the Department of Dermatology and Allergy of Hannover Medical School (MHH). The work described was carried out in accordance with the Code of Ethics of the World Medical Association (Declaration of Helsinki), and patients gave their written informed consent prior to the study.

### Staphylococcus aureus

Wild-type (strain SH1000) and methicillin-resistant (strain MW2) *S. aureus* were grown in Luria broth (LB, Oxoid, Germany), and aliquots of log-phase cultures were stored at −80°C. For infection of cells, fresh log-phase cultures were prepared from frozen stock.

### THP-1 cells

The THP-1 cells (clone TIB-202) were obtained from the American Type Culture Collection (ATCC, Manassas, VA, USA). The cells were cultured in a Roswell Park Memorial Institute (RPMI) cell culture medium supplemented with 10% fetal calf serum (FCS) and 1% penicillin/streptomycin.

### Generation of *CYLD*-deficient THP-1 cells

*CYLD* was knocked down by the CRISPR/Cas9 method using SG cell line 4D-nucleofector X kit (#V4XP-4024, Lonza, Basel, Switzerland). For 1 × 10^6^ cells, 210 pmol of duplexed gRNA was mixed with 70 pmol of Cas9 Nuclease V3 (#1081059, IDT, Coralville, IA, USA) for RNP complex synthesis, and the complex was mixed with 70 pmol of EE buffer (#1075916, IDT) to form electroporation mix. Electroporation was performed using the preset program DZ100 in the Lonza nucleofection system.

### Generation of macrophages from THP-1 cells

THP-1 cells were differentiated into macrophages through Phorbol 12-myristate 13-acetate (PMA) (50 ng/mL) treatment for 48 h. The macrophages were either maintained in an unpolarized state (M0) or polarized into the M1 phenotype by stimulation with IFN-γ (20 ng/mL) and lipopolysaccharide (LPS) (10 pg/mL).

### Generation of monocyte-derived macrophages

Peripheral blood mononuclear cells (PBMCs) were isolated using Ficoll density gradient centrifugation followed by magnetic cell separation of CD14^+^ monocytes (#480094, MojoSort, BioLegend, San Diego, CA, USA). CD14^+^ monocytes were then cultured for 6 days in Dulbecco’s modified Eagle medium (DMEM) medium supplemented with 50 ng macrophage colony-stimulating factor (M-CSF). Cells were harvested and stimulated as per experimental requirements. MDMs at day 7 polarized into M1 phenotype by stimulation with IFN-γ (20 ng/mL) and LPS (10 pg/mL).

### *In vitro* infection of cells with *S. aureus*

*In vitro* infection of THP-1 macrophages and MDMs was performed as previously described (38). THP-1 macrophages and MDMs were infected with *S. aureus* at a multiplicity of infection (MOI) of 1:1. The dose of infection was verified by plating on Luria Broth (LB) agar. One hour post-infection, the extracellular bacteria were killed by incubating the cells with 30 µg/mL gentamicin (Sigma-Aldrich, St. Louis, MO, USA) for 30 min. The cells were thereafter washed with phosphate-buffered saline (PBS) to remove the extracellular bacteria and further cultivated in a medium containing gentamicin for the indicated time points. NF-κB was inhibited in THP-1 macrophages by treating the cells with IKK inhibitor VII (1 µM; Calbiochem, Darmstadt, Germany) for 24 h before the infection.

### CFUs

The bacterial load in infected THP-1 macrophages and MDMs was enumerated as previously described (38). In brief, 24 h p.i., *S. aureus*-infected cells were washed twice with PBS to remove the antibiotics, the cells were then lysed with 0.1% Triton X-100, and serial dilution was made and plated on LB agar. Bacterial colonies were enumerated microscopically after incubation at 37°C for 24 h and 48 h.

### Protein isolation and Western blotting

Proteins were isolated from *S. aureus*-infected THP-1 macrophages as previously described (38). In brief, cells were lysed using the lysis buffer (38). The lysates were centrifuged to remove the cell debris and the protein for Western blotting. For the Western blotting, equal amounts of proteins were separated on sodium dodecyl sulfate (SDS)–polyacrylamide gels as described previously (38), and the proteins were then transferred onto polyvinylidene fluoride membranes. To block the non-specific binding of antibodies, the membrane was incubated either with Blotto B [1% nonfat dry milk + 1% bovine serum albumin (BSA)], 5% nonfat dry milk, or 5% BSA for 1 h. The proteins were stained for GAPDH, phospho-STAT1 Y701, phospho-STAT1 S727, STAT-1, MyD88, IRAK-4, phospho-IRAK-4, TRAF6, NOD2, phospho-RIPK2, RIPK2, p65phospho-p65, p65, phospho-p38, p38, phospho-ERK1/2, ERK1/2, phospho-JNK, JNK, IKKγ/NEMO, CYLD, and K63-linkage-specific polyubiquitin overnight (all antibodies were obtained from Cell Signaling Technology, Frankfurt, Germany). The following day, membranes were washed using Tris-buffered saline with 0.1% Tween 20 (TBST) incubated with anti-mouse or anti-rabbit secondary antibodies (Dako, Hamburg, Germany) for 1 h. The blots were washed in TBST and developed using an ECL Plus kit (GE Healthcare, Freiburg, Germany). Western blotting (WB) images were captured using the Intas Chemo Cam Luminescent Image Analysis system^®^ (INTAS Science Imaging Instruments, Göttingen, Germany) and analyzed using the LabImage 1D software^®^ (Kapelan Bio-Imaging Solutions, Leipzig, Germany) (38).

### Immunoprecipitation

Immunoprecipitation of CYLD and STAT1 was performed as described previously (38). In brief, proteins from uninfected and *S. aureus*-infected THP-1 macrophages were lysed using the lysis buffer as described. The protein samples were precleared using Gamma Bind™ G Sepharose™ beads (GE Healthcare Europe GmbH, Freiburg, Germany) to remove the proteins that non-specifically bind to the beads. The proteins were thereafter incubated with anti-STAT1 (1:100) or anti-CYLD (1:100) antibodies at 4°C overnight. IgG antibody was used as a negative control. The protein antibody complex was precipitated using fresh Gamma Bind™ G Sepharose™ beads at 4°C overnight. The protein antibody complex was then washed and incubated with 1× lane marker reducing sample buffer and heated at 99°C for 5 min. Thereafter, samples were centrifuged, and the supernatant was used to detect STAT1, CYLD, K63, TRAF6, RIPK2, IKK/NEMO, and K63-linked ubiquitin by WB. GAPDH was used as the input control (38).

### Measurement of NO

The production of NO_2_ by *S. aureus*-infected THP-1 macrophages was determined using the Griess Assay Kit (Promega, Mannheim, Germany) as previously described (38). In brief, the supernatant from infected and non-infected cells was incubated first with sulfanilamide solution and thereafter with *N*-(1-naphthyl)ethylenediamine solution in the dark for 10 min. The concentration of NO_2_ was determined using a Synergy^®^ microplate reader (Biotek, Berlin, Germany).

### ROS detection

ROS in *S. aureus*-infected THP-1 macrophages were determined using a ROS detection kit (Enzo Life Sciences, Lörrach, Germany) as described previously (38). In brief, *S. aureus*-infected THP-1 macrophages were washed twice with the washing buffer. Thereafter, the cell pellet was resuspended in the detection reagent and incubated in the dark at 37°C for 30 min. The samples were analyzed by Cytek Aurora flow cytometry (Fremont, CA, USA).

### LDH assay

Lactate dehydrogenase (LDH) enzyme activity was determined in *S. aureus*-infected THP-1 macrophages using CyQUANT LDH Cytotoxicity Assay (#C20300, Thermo Fisher Scientific, Waltham, MA, USA) according to the manufacturer’s protocol.

### CRISPR/Cas9 knockout of CYLD

Stable knockout of *CYLD* was generated by the CRISPR/Cas9 system using the gRNA Hs.Cas9.CYLD.1.AA:TCACTGACGGGGTGTACCAA and SG cell line 4D-nucleofector X kit (#V4XP-3024, Lonza) according to the manufacturer’s protocol.

### *In vitro* siRNA treatment

For siRNA-mediated knockdown of STAT1, WT and *CYLD* ^−/−^ THP-1 macrophages were transfected with 20 nm of STAT1-specific siRNA (Dharmacon, Lafayette, CO, USA) according to the manufacturer’s instruction. Scrambled siRNA at 20 µM was used as a control. A total of 50 µL of the siRNA mixture was added to each well in a 24-well plate containing 4 × 10^5^ macrophages and incubated at 37°C for 48 h. The efficiency of siRNA-mediated STAT1 silencing was controlled by WB.

### Immunostaining

OCT-embedded tissues were cut into 6-mm cryosections, air-dried, and fixed at 4°C in acetone for 10 min. After washing, horseradish peroxidase (HRP) and Alkaline Phosphatase (AP) blocking solution was applied for 20 min (Invitrogen, Waltham, MA, USA), followed by Superblock (Thermo Scientific, Waltham, MA, USA). Rabbit polyclonal anti-CYLD (ab33929, Abcam, Cambridge, UK) or rabbit Ig (Dako X 0936, Agilent, Santa Clara, CA, USA) were applied in equal concentrations. Mouse monoclonal anti-CD3 (Agilent) was used to stain the cellular skin infiltrate. After applying the Envision+ HRP conjugate (Agilent) with anti-rabbit K4009 and anti-mouse K005, the AEC substrate kit was applied (AEC, 3-amino-9-ethylcarbazole; Zytomed Systems, Bargteheide, Germany). Images were taken using a Pannoramic MIDI II Sola (Sysmex, Norderstedt, Germany).

### Single-cell RNA sequencing

Data from a previous study that included single-cell RNA sequencing were used for the investigation of CYLD expression on a single-cell level (Zhang et al., 2023). For a comprehensive explanation, refer to the corresponding methods section of the study. Briefly, skin punch biopsies were processed using the Miltenyi Biotech skin dissociation kit for subsequent cell isolation and CD2 enrichment, followed by pooling and loading onto the chromium chip (10x Genomics, Pleasanton, CA, USA). The CellRanger pipeline version 3.1.0 was employed to align reads to the human reference genome GRCH38. The expression matrix generated was then analyzed using the Seurat package version 4.2.3. UMAP and feature plots were generated using their respective functions.

### Statistics

The statistical significance was determined using the software Prism 9 with respective tests as mentioned in the figure legends, and p-values of ≤0.05 were considered significant. All experiments were performed at least twice.

## Results

### CYLD expression in eczematous skin lesions of AD patients

**Figure 1:**
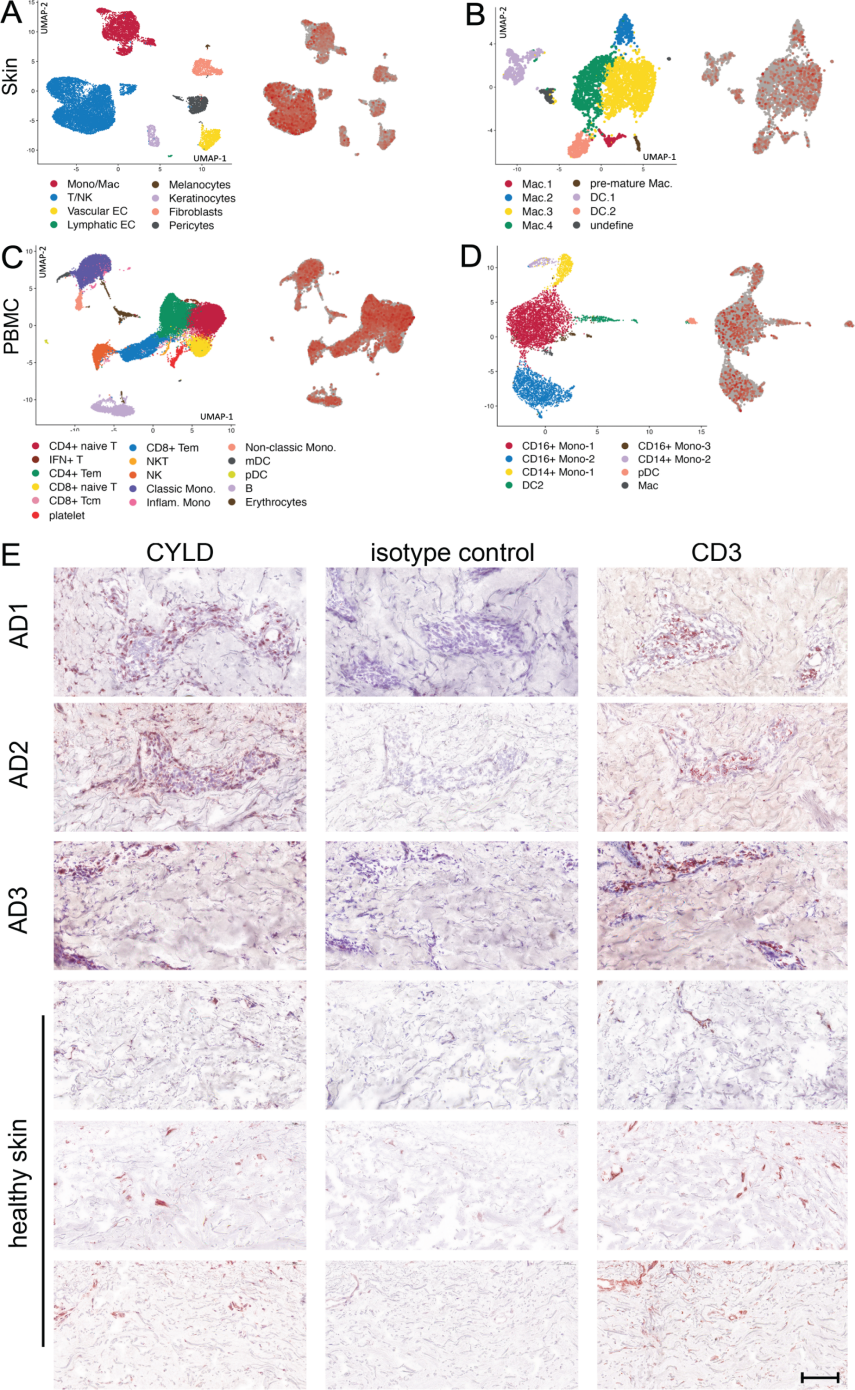
CYLD mRNA and protein expression in atopic dermatitis (AD). **(A–D)** Lesional skin and peripheral blood mononuclear cells (PBMCs) of 10 patients with AD were investigated by scRNA-seq as reported earlier (Zhang et al., 2023). Expression of CYLD in skin-derived leukocytes **(A)** and myeloid cell populations **(B)** as well as PBMCs **(C)** and myeloid cells thereof **(D)** is visualized by red color on the right dot plot of each panel. **(E)** Exemplary pictures of CYLD protein expression (brown, AEC) in the skin of three patients with AD and three healthy control donors. The eczematous lesion of patient AD3 showed signs of superinfected eczema. Sections were immunostained with a polyclonal rabbit anti-CYLD antibody or respective isotype control and counterstained with hemalum. Mouse anti-CD3 was applied as a reference. Scale bar = 100 μm.

The expression of CYLD in different organs and cells under homeostatic conditions has been summarized in “The Human Protein Atlas” (https://www.proteinatlas.org/ENSG00000083799-CYLD). These data show that CYLD is expressed in all organs including the skin, with strong protein and mRNA expression in keratinocytes, Langerhans cells, and immune cells including T cells, macrophages, and B cells but not in melanocytes (43). CYLD is expressed in all leukocyte populations including granulocytes and monocytes (https://www.proteinatlas.org/ENSG00000083799-CYLD/immune+cell). Transcriptome analysis of skin biopsies from 10 AD patients identified CYLD mRNA in T cells, natural killer cells (**Figure 1A**), and different macrophage subtypes including M2 and dendritic cells (DCs) (**Figure 1B**). mRNA expression in keratinocytes, fibroblasts, vascular endothelial cells, and pericytes appeared weaker but was present as well, while CYLD expression was also detectable in PBMC samples of the same donors including different myeloid cells (**Figures 1C, D**). Our histological analysis of CYLD expression in AD patients revealed the expression of CYLD in CD3^+^ T cells as well as non-T cells (**Figure 1E**). CYLD expression appeared particularly strong in the lesional skin of a patient with clinical signs of superinfected eczema (**Figure 1E**, patient AD3). Our further histological analysis confirmed the widespread CYLD expression in the cellular infiltrate and keratinocytes of inflamed AD skin lesions as compared to healthy skin (**Supplementary Figure S1**). Thus, CYLD is expressed in the skin of both healthy persons and AD patients with expression in identical cell types including skin macrophages and monocytes in the blood.

### Increased CYLD expression and impaired control of *S. aureus* in monocyte-derived macrophages of AD patients

**Figure 2:**
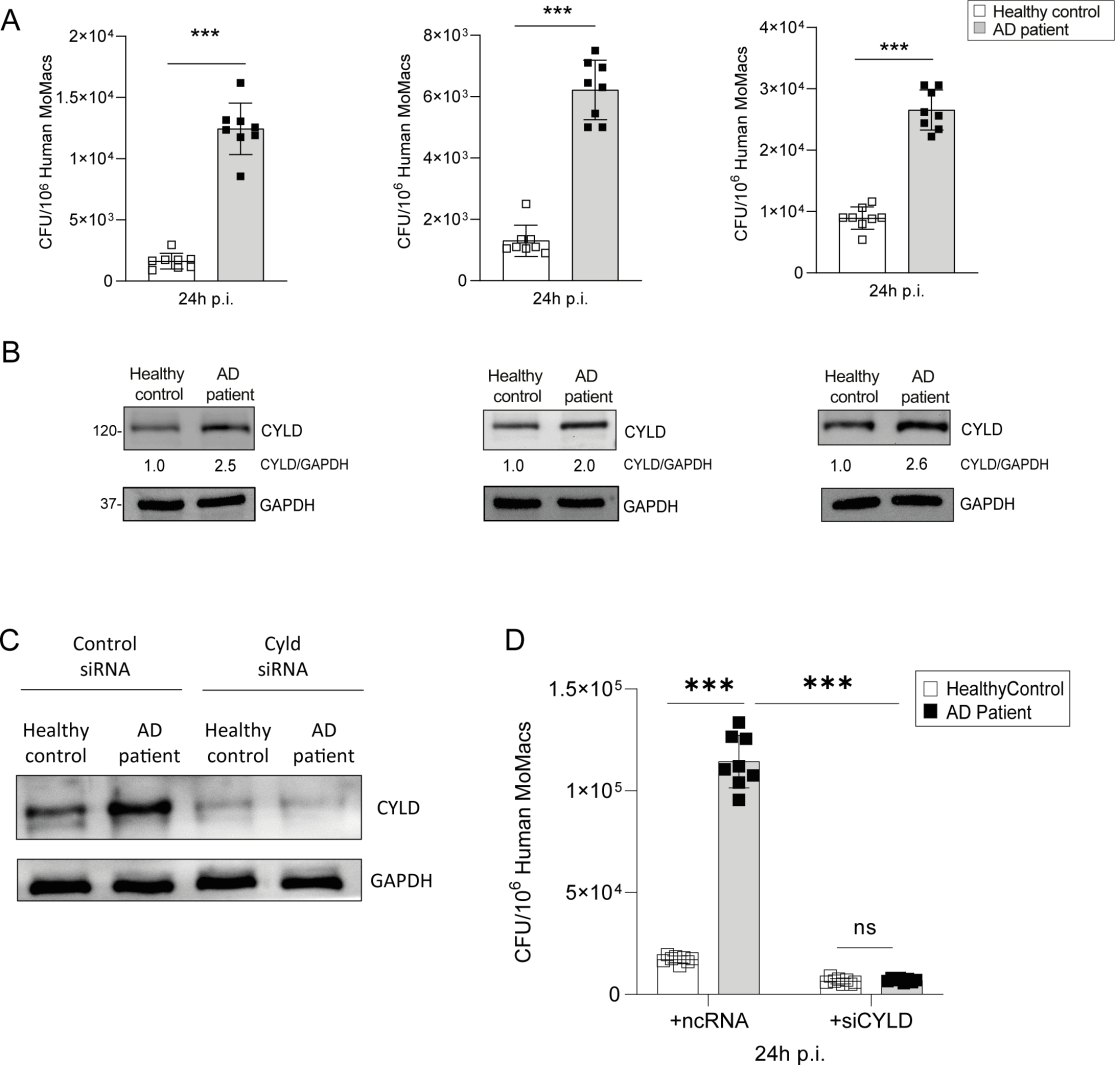
Elevated CYLD expression and impaired control of *Staphylococcus aureus* by monocyte-derived macrophages from atopic dermatitis patients. CD14^+^ monocytes were isolated from the blood of atopic dermatitis patients and healthy controls using density gradient centrifugation followed by Magnetic-activated cell sorting (MACS) for CD14^+^ cells; thereafter, the monocytes were differentiated into macrophages through macrophage colony-stimulating factor (M-CSF) (50 ng/mL) treatment. **(A)** M1-polarized macrophages from atopic dermatitis patients and healthy controls were infected with *S. aureus* [strain MW2; multiplicity of infection (MOI) 1:1] for 24h. Thereafter cells were lysed, serial dilutions of the cell lysates were plated on agar plates, and the bacterial colonies were enumerated after 24 h (n = 8 per group). **(B)** The macrophages from healthy control and atopic dermatitis (AD) patients were polarized into M1 phenotype by stimulation with IFN-γ (20 ng/mL) and lipopolysaccharide (LPS) (10 pg/mL) for 24h. Cells were harvested, and protein lysates were analyzed for CYLD expression by Western blotting. GAPDH was used as a loading control. **(C, D)** CD14^+^ monocytes were isolated from buffy coats of healthy controls and AD patients by density gradient centrifugation and MACS and differentiated into macrophages by M-CSF (50 ng/mL) treatment. Thereafter, cells were transfected with CYLD siRNA or control siRNA. Twenty-four hours after transfection, monocyte-derived macrophages (MDMs) were polarized into M1 by stimulation with IFN-γ (20 ng/mL) and LPS (10 pg/mL) for 24h. **(C)** The efficiency of the CYLD knockdown was analyzed 48 h after transfection by Western blotting (WB). **(D)** After stimulation for 24 h, MDMs were infected with *S. aureus* (strain MW2; MOI 1:1). The intracellular bacterial load was determined at 24 h p.i. (n = 8 per group). Bars represent mean values ± SD (Student’s unpaired t-test, **p < 0.01 ). Data from one of two independent experiments with similar results are shown. ***p<0.001, ns, non-significant. Bars represent mean values ±SD (Student`s unpaired t-test, ***p<0.001).

Since AD is characterized by a type 2 inflammation including M2 macrophage polarization (19) and activation of the IFN-γ/STAT1 and TLR2/4 pathways synergize in the control of *S. aureus*, we first determined whether M1 polarization of macrophages improves the control of *S. aureus*-infected macrophages of AD patients to the same extent as in healthy controls. In these experiments, we isolated CD14^+^ monocytes from the blood of AD patients and healthy controls, and we differentiated these cells into macrophages by M-CSF treatment followed by IFN-γ/LPS treatment. Control of *S. aureus* was significantly impaired in MDMs from all investigated AD patients (**Figure 2A**). Since CYLD can impair the cell-intrinsic control of intracellular bacteria (37, 38), we determined the CYLD expression in the infected MDMs. WB analysis showed that the impaired capacity of M1-polarized MDMs of AD correlated with an increased CYLD protein expression in these infected cells (**Figure 2B**).

Thus, AD patients are characterized by an overexpression of CYLD in macrophages and impaired control of *S. aureus*. To corroborate the inhibitory function of CYLD on the control of *S. aureus* in M1-polarized macrophages of AD patients, we deleted CYLD by siRNA in IFN-γ/LPS-primed (M1) macrophages of healthy blood donors and AD patients (**Figure 2C**). Upon infection, the control of *S. aureus* was significantly improved in CYLD-deleted macrophages of AD patients as compared to control siRNA-treated macrophages (**Figure 2D**). Thus, CYLD is upregulated in MDMs of AD patients and impairs the control of *S. aureus* in M1-polarized MDMs, demonstrating that CYLD is a macrophage intrinsic inhibitor of the control of *S. aureus*.

### CYLD inhibits M1 macrophage polarization, production of anti-bacterial reactive oxygen species, and control of *S. aureus*

**Figure 3:**
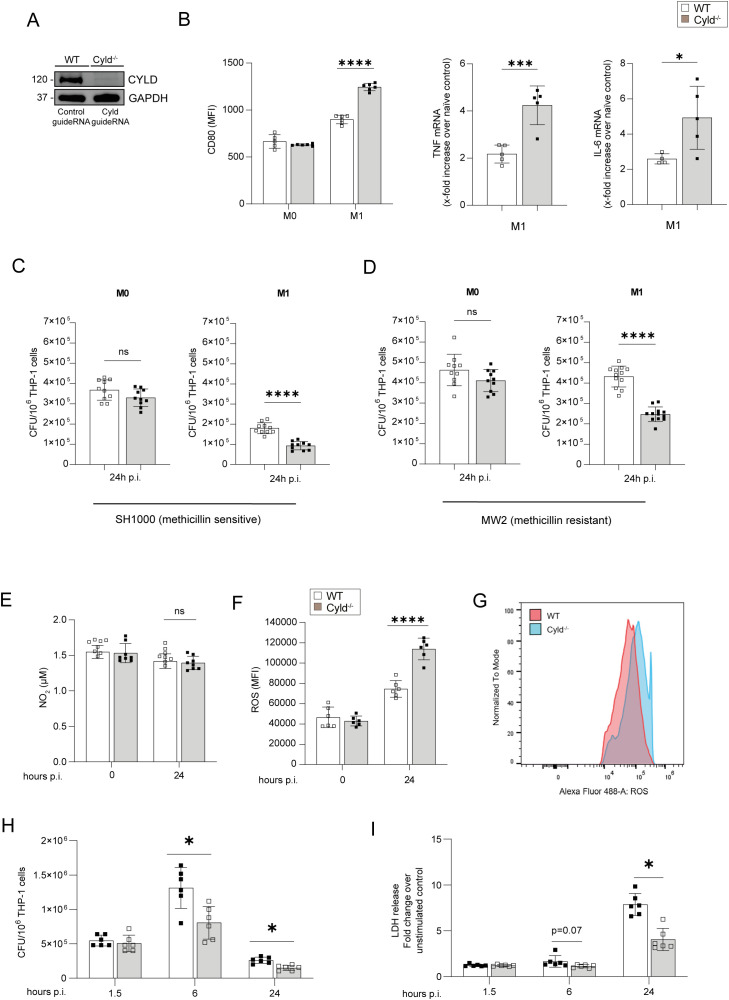
CYLD impairs M1 polarization of macrophages, fosters intracellular control of *Staphylococcus aureus* in THP-1-derived M1 macrophages, and impairs TNF and reactive oxygen species (ROS) production in M1 macrophages upon *S. aureus* infection. **(A)** *CYLD* was deleted in THP-1 cells by CRISPR/Cas9 and subsequently differentiated into macrophages through PMA (50 ng/mL) treatment. The efficacy of the CYLD knockout was evaluated via Western blotting (WB) analysis. GAPDH was utilized as a loading control. Wild type (WT) and *CYLD*-deficient (*CYLD* ^−/−^) THP-1-derived macrophages were polarized into M1 phenotype by stimulation with IFN-γ (20 ng/mL) and lipopolysaccharide (LPS) (10 pg/mL) for 24 h or left unpolarized (M0). **(B)** Twenty-four hours after stimulation, macrophages were harvested and stained for the M1 marker CD80. Cells were fixed with 4% Paraformaldehyde (PFA), and the mean fluorescence intensity (MFI) was measured by flow cytometry (n = 5 per group). A quantitative RT-PCR (qRT-PCR) analysis of signature cytokines of M1 macrophages TNF and IL-6 mRNA expression was performed 24 h after stimulation (n = 6 per group). Changes in gene expression were normalized to Hypoxanthine-guanine phosphoribosyltransferase (HPRT). In panels A and B, bars represent mean values ± SD (Student’s unpaired t-test, *p < 0.05, ***p < 0.001). **(C, D)** Wild-type (WT) and *CYLD*-deficient (*CYLD* ^−/−^) THP-1-derived macrophages were polarized into the M1 phenotype by stimulation with IFN-γ (20 ng/mL) and LPS (10 pg/mL) for 24 h, followed by infection with *S. aureus* [multiplicity of infection (MOI) 1]. The intracellular bacterial load was determined in unpolarized M0 and polarized M1 wild-type (WT) and Cyld^−/−^ macrophages 24 h post-infection (p.i.) with **(C)** methicillin-sensitive *S. aureus* (strain SH1000) and **(D)** methicillin-resistant *S. aureus* (strain MW2) (n = 10 per group). The cells were lysed, serial dilutions of the cell lysates were plated on agar plates, and the bacterial colonies were enumerated after 24h. In panels A and B, the bars represent the mean values ± SD (Student’s unpaired t-test, ***p < 0.001, ****p < 0.0001). The data presented are from one of two independent experiments with similar results. **(E–G)** Wild-type (WT) and *CYLD*-deficient (*CYLD* ^−/−^) THP-1-derived macrophages were polarized into the M1 phenotype by stimulation with IFN-γ (20 ng/mL) and LPS (10 pg/mL) for 24h. Thereafter, cells were infected with *S. aureus* (strain MW2; MOI 1:1). **(E)** The NO_2_ concentration in the supernatant of uninfected (0 h) and infected (24 h p.i.) WT and Cyld^−/−^ macrophages was measured photometrically by the Griess assay (n = 8 per group). **(F, G)** The level of intracellular ROS was determined at (0 h) and 24 h p.i. by flow cytometry using a ROS detection kit (n = 6 per group). **(H)** The intracellular bacterial load was determined in M1-polarized wild-type (WT) and Cyld^−/−^ macrophages at 1.5, 6, and 24 h p.i. with methicillin-resistant *S. aureus* (strain MW2) (n = 6 per group). **(I)** Cell death was determined at 1.5, 6, and 24 h p.i by measuring lactate dehydrogenase enzyme activity using lactate dehydrogenase (LDH) cytotoxicity assay kit (n = 6 per group). In panels B–I, bars represent mean values ± SD (Student’s unpaired t-test, *p < 0.05, ****p < 0.0001, ns, non-significant). Data from one of two independent experiments with similar results are shown.

To determine the mechanisms of how CYLD impairs intrinsic immunity to *S. aureus*, we established *CYLD*-deficient THP-1 monocytes by CRISPR/Cas9 (**Figure 3A**). Upon PMA-mediated differentiation into macrophages and subsequent IFN-γ/LPS stimulation, *CYLD*-deficient THP-1 cells expressed higher levels of CD80, a marker for M1 macrophages, and TNF and IL-6 mRNA, two prototypic M1 macrophage cytokines important for the control of *S. aureus* (**Figure 3B**). In IFN-γ/LPS-stimulated THP-1 macrophages, *CYLD* deletion significantly improved control of methicillin-sensitive and methicillin-resistant *S. aureus*, whereas no differences between the two genotypes were observed for unstimulated macrophages (**Figures 3C, D**). Analysis of anti-bacterial nitric oxide and reactive oxygen species revealed that ROS were significantly increased in infected *CYLD*-deficient THP-1 24 h p.i., whereas NO levels did not differ (**Figures 3E–G**). A kinetic analysis of bacterial loads revealed that CYLD does not influence the entry of *S. aureus* into the macrophages (**Figure 3H**) but impairs intracellular bacterial control, which leads to the death of macrophages (**Figure 3I**). These data illustrate that CYLD impaired M1 macrophage differentiation, control of *S. aureus*, and ROS production.

### CYLD inhibits STAT1, MyD88, and NOD2 signaling in *S. aureus*-infected M1-polarized macrophages

Since M1-polarized macrophages showed improved control of *S. aureus* and anti-bacterial activity in *CYLD*-deficient macrophages, we determined the impact of *CYLD* on i) IFN-γ-induced STAT1 activation, ii) the effect of LPS (TLR4) and *S. aureus* (TLR2)-induced MyD88 signaling, and iii) *S. aureus*-activated NOD2 pathways.

**Figure 4:**
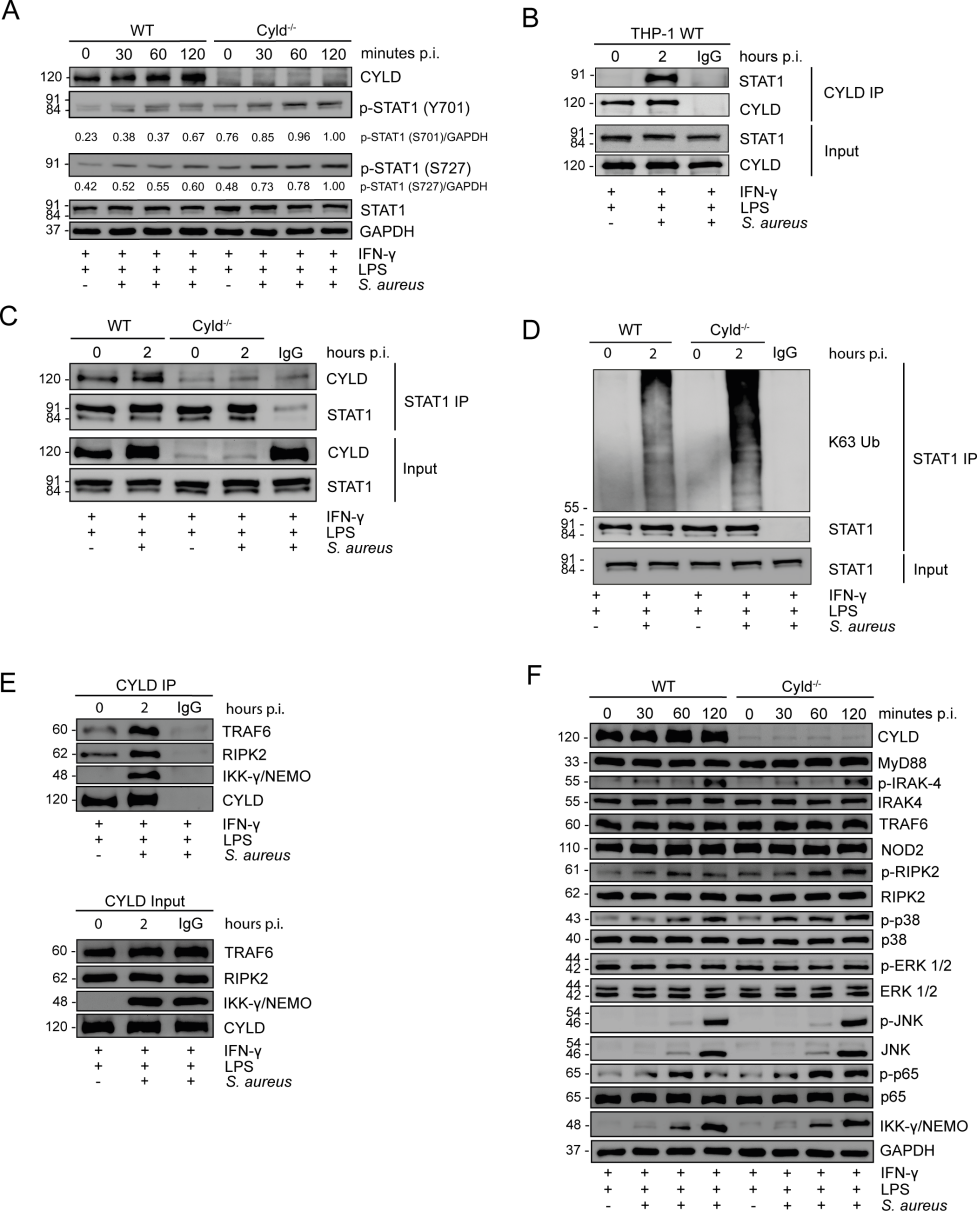
CYLD impairs STAT1, NF-κB, and pathways upon *Staphylococcus aureus* infection. **(A–C)** Wild type (WT) and *CYLD* ^−/−^ THP-1-derived macrophages were polarized into M1 phenotype by stimulation with IFN-γ (20 ng/mL) and lipopolysaccharide (LPS) (10 pg/mL) for 24h. Thereafter, cells were infected with *S. aureus* [strain MW2; multiplicity of infection (MOI) 5:1] and harvested at 0 (uninfected), 30, 60, and 120 minutes p.i. Proteins were isolated, and a Western blotting analysis of the indicated proteins was performed. GAPDH was used as a loading control. Representative Western blotting from one of two independent experiments is shown. **(A)** WT and *CYLD* ^−/−^ THP-1-derived macrophages were polarized into M1 by stimulation with IFN-γ (20 ng/mL) and LPS (10 pg/mL) for 24h. Thereafter, cells were infected with *S. aureus* (strain MW2; MOI 5:1) and harvested at 0 (uninfected), 30, 60 and 120 minutes p.i. Proteins were isolated, and a Western blotting analysis of the indicated proteins was performed. GAPDH was used as a loading control. Representative Western blotting from one of two independent experiments is shown. **(B)** THP-1-derived macrophages were polarized into M1 by stimulation with IFN-γ (20 ng/mL) and LPS (10 pg/mL) for 24 h, followed by infection with *S. aureus* (strain MW2; MOI 5:1). Two hours after infection, cells were harvested, and proteins were isolated. A co-immunoprecipitation with CYLD antibody was performed, and immunoprecipitates were stained for CYLD and STAT1. **(C, D)** WT and Cyld^−/−^ THP-1-derived macrophages were polarized into M1 by stimulation with IFN-γ (20 ng/mL) and LPS (10 pg/mL) for 24 h followed by infection with *S. aureus* (strain MW2; MOI 5:1). Two hours after infection, cells were harvested, and protein lysates were co-immunoprecipitated with STAT1 antibody. Western blotting analysis for CYLD **(C)**, STAT1 **(C, D)**, and K63-linked polyubiquitin **(D)** was performed. In panels B–D, representative Western blotting from one of three independent experiments is shown. **(E)** WT and *CYLD* ^−/−^ THP-1-derived macrophages were polarized into M1 by stimulation with IFN-γ (20 ng/mL) and LPS (10 pg/mL) for 24 h followed by infection with *S. aureus* (strain MW2; MOI 5:1). Two hours after infection, cells were harvested, and protein lysates were co-immunoprecipitated with CYLD antibody. Western blotting analysis for CYLD, RIPK2, and IKKγ/NEMO was performed. **(F)** WT and *CYLD* ^−/−^ THP-1-derived macrophages were polarized into M1 phenotype by stimulation with IFN-γ (20 ng/mL) and LPS (10 pg/mL) for 24h. Thereafter, cells were infected with *S. aureus* (strain MW2; MOI 5:1) and harvested at 0 (uninfected), 30, 60 and 120 minutes p.i. Proteins were isolated, and a Western blotting analysis of the indicated proteins was performed. GAPDH was used as a loading control.

WB analyses showed that *CYLD* impaired STAT1 phosphorylation at tyrosine 701 and serine 727, which cooperatively led to the induction of IFN-γ-induced genes (Sadzak et al., 2008; Varinou et al., 2003) (**Figure 4A**). Co-immunoprecipitation experiments newly identified that *CYLD* directly interacted with STAT1 (**Figures 4B, C**). STAT1 immunoprecipitation and subsequent WB analysis of K63 ubiquitin revealed that *S. aureus* infection induced increased K63 ubiquitination of STAT1 in *CYLD*-deficient macrophages as compared to WT macrophages at 2 h p.i. (**Figure 4D**). In agreement with published data in other models (29, 30, 32, 38), *CYLD* also interacted with TRAF6, IKKγ/NEMO, and RIPK2 in M1-polarized *S. aureus*-infected THP-1 macrophages (**Figure 4E**). This was associated with impaired MyD88 and NOD2/RIPK2 signaling, leading to reduced downstream NF-κB activation shown by impaired phosphorylation of p65 in *S. aureus*-infected *CYLD*-competent macrophages (**Figure 4F**). Thus, *CYLD* inhibits simultaneously key pathways leading to reduced activation of the transcription factors NF-κB and STAT1.

### The enhanced control of intracellular *S. aureus* in *CYLD*-deficient THP-1-derived M1 macrophages is NF-κB- and STAT1-dependent

**Figure 5:**
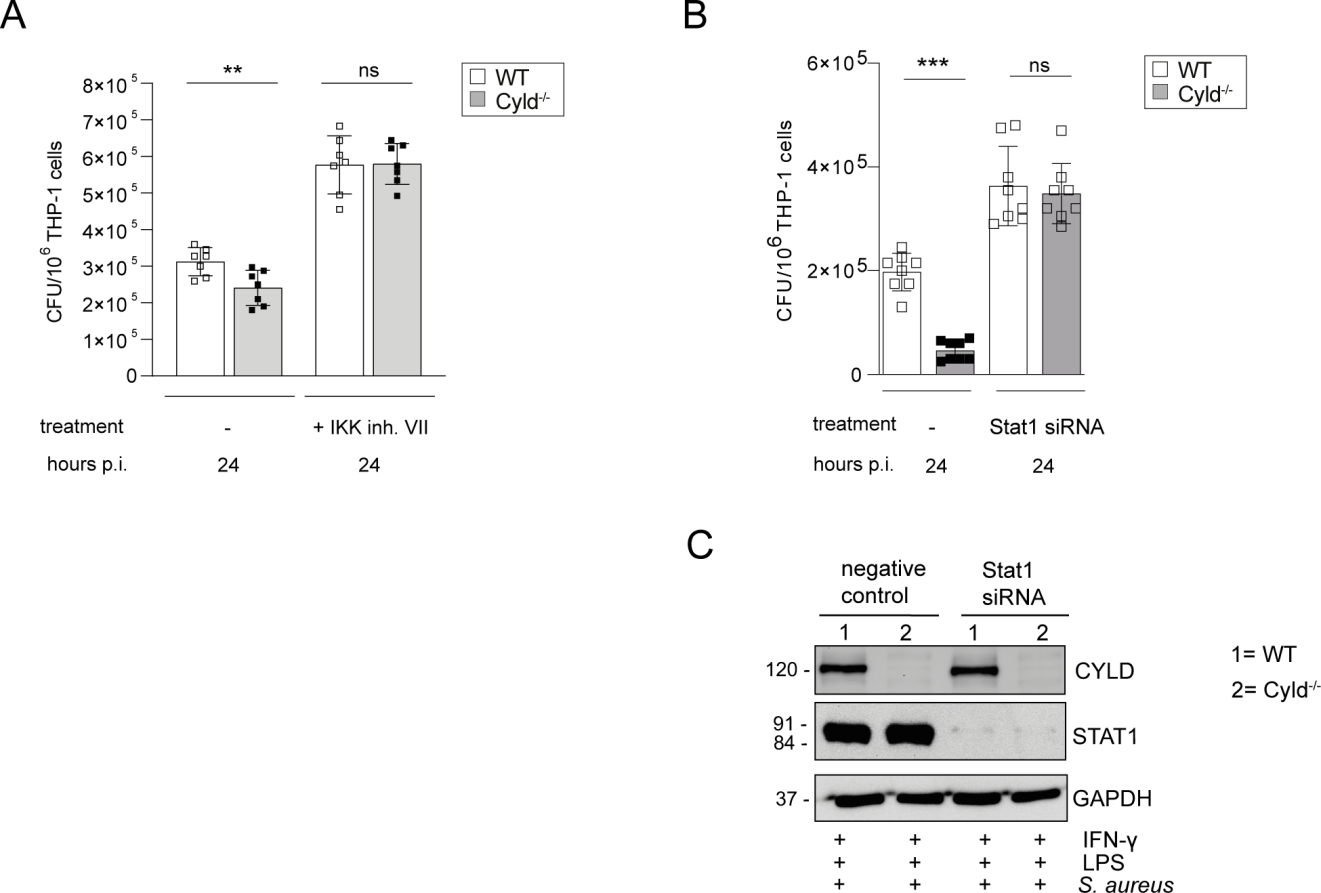
The improved control of intracellular *Staphylococcus aureus* in Cyld-deficient THP-1-derived M1 macrophages is NF-κB- and STAT1-dependent. WT and Cyld^-/-^ THP-1-derived macrophages were polarized into M1 phenotype by stimulation with IFN-γ (20 ng/ml) and LPS (10 pg/ml) for 24 h. **(A)** Cells were either left untreated or treated with IKK inhibitor VII (1 μM), thereafter, the cells were infected with *S. aureus* (strain MW2, MOI 1:1). The inhibitor treatment was continued during and after infection. Twenty-four hours p.i., the intracellular bacterial load of the different groups of methicillin-resistant *S. aureus* (MRSA)-infected WT and Cyld^−/−^ macrophages was determined (n = 6 per group). **(B)** For STAT1 knockdown, STAT1 siRNA was added to WT and *CYLD* ^−/−^ macrophages 24 h prior to addition of IFN-γ (20 ng/mL) and LPS (10 pg/mL) followed by infection with *S. aureus* strain MW2 (MOI 1:1). The intracellular bacterial load was determined 24 h p.i. **(C)** Efficiency of STAT1 knockdown was validated using Western blotting. Bars represent mean values ± SD (Student’s unpaired t-test, **p < 0.01, ***p<0.001, ns, non-significant). Data from one of two independent experiments with similar results are shown.

To determine the functional importance of *CYLD*-regulated NF-κB and STAT1 activation for the control of *S. aureus*, we stimulated THP-1 macrophages with IFN-γ/LPS for M1 polarization and treated the cells prior to infection with the IKK VII inhibitor to inhibit activation of NF-κB and with STAT1 siRNA to inhibit STAT1. Inhibition of NF-κB activation and STAT1 resulted in a strong increase of colony-forming units (CFUs) of *S. aureus* in both WT and *CYLD*-deficient THP-1 macrophages, and both treatments abolished the differences between the two genotypes (**Figures 5A, B**). Thus, the inhibition of both STAT1 and NF-κB by *CYLD* is critical for the control of *S. aureus* in macrophages.

### *Cyld*-deficient mice are protected from *S. aureus* infection

**Figure 6:**
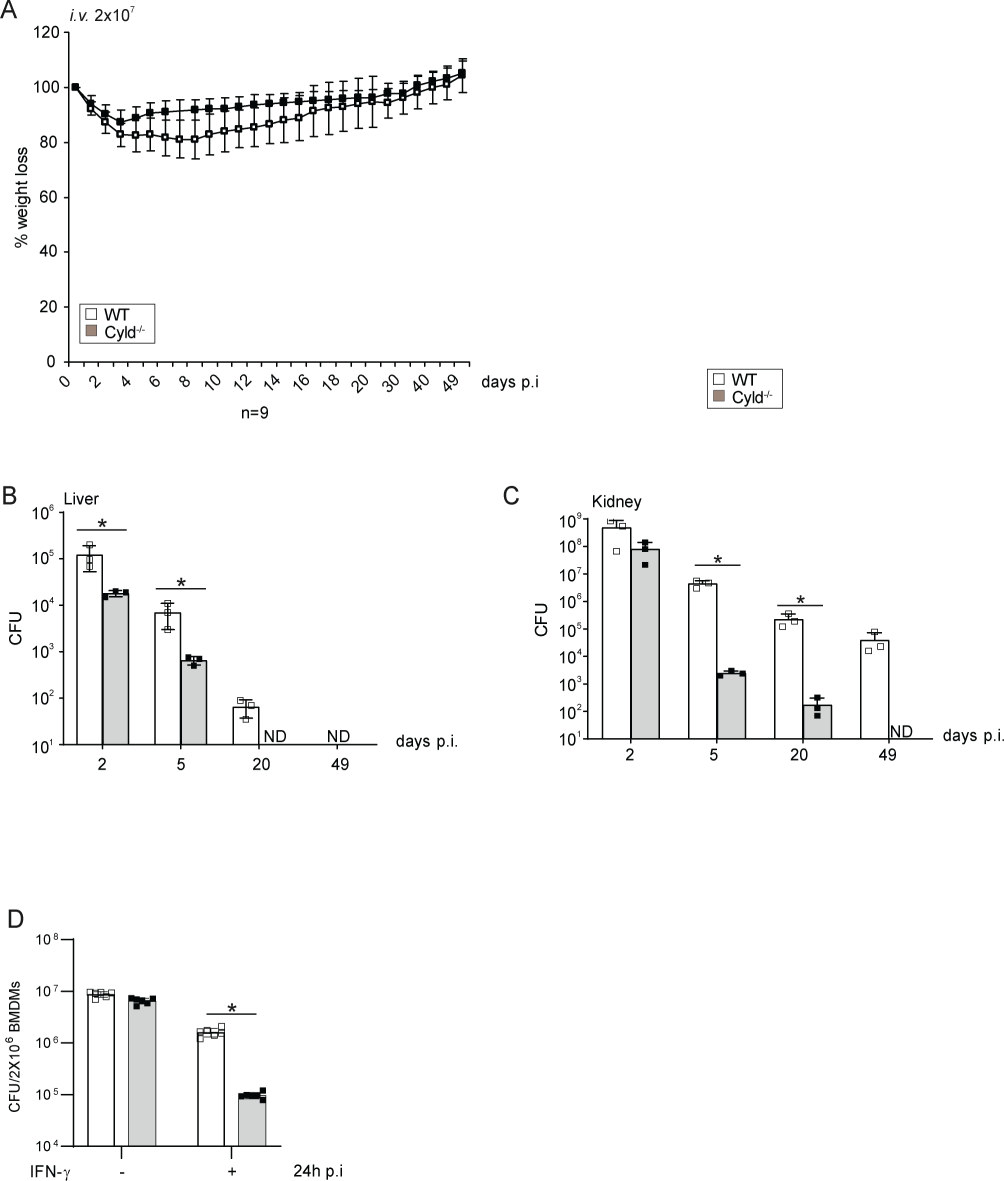
CYLD-deficient mice are protected from *Staphylococcus aureus* infection. **(A–C)** C57BL/6 wild type (WT) and Cyld^−/−^ mice (8–10 weeks old) were intravenously infected with 2 × 10^7^ *S. aureus* (strain SH1000). **(A)** Weight of mice was monitored daily up to day 50 p.i. The weight on the day of infection was defined as 100%, and the percentage of differences in weight compared to the 100% are shown (n = 6–8 mice per group). **(B, C)** Mice were sacrificed at the indicated timepoints p.i., and 10-fold serial dilutions of organ homogenates were plated on agar plates. After 24 h, colonies were counted microscopically, and the colony-forming units (CFUs) were calculated for liver **(B)** and kidney **(C)** (n = 6 per timepoint and group). **(D)** Triplicates of bone marrow-derived macrophages (BMDMs) were treated with IFN-γ (100 U/mL) for 6 h before infection with *S. aureus* in a gentamicin protection assay [multiplicity of infection (MOI) 5:1] for 24 h Thereafter, cells were lysed, and CFUs were determined. In panels **A–D**, the mean values ± SD are shown (Student’s unpaired t-test, *p < 0.05). Data from one of two independent experiments with similar results are shown.

The data presented identify that CYLD is increasingly expressed in macrophages of AD patients and inhibits the control of *S. aureus* in human macrophages. To further validate an inhibitory function of CYLD in *S. aureus* infection and to evaluate whether systemic CYLD inhibition may be a therapeutic option to ameliorate *S. aureus* infection, we infected *Cyld*-deficient and WT mice with *S. aureus*. *Cyld*-deficient mice had significantly reduced weight loss (**Figure 6A**) and improved pathogen control in the liver and kidney at the acute and chronic stages of infection (**Figures 6B, C**). In good agreement with the human macrophages, CYLD-deficient murine bone marrow-derived macrophages showed better clearance of *S. aureus* compared to WT macrophages (**Figure 6D**). This qualifies CYLD as a relevant therapeutic target to ameliorate *S. aureus* infections.

## Discussion

Macrophages play a fundamental role in the control of local and systemic infections with *S. aureus* (12, 13, 45). In murine *S. aureus* skin infection, M1-polarized macrophages contribute to the control of the infection, whereas M2-polarized macrophages are associated with impaired killing of *S. aureus* and spread of the pathogen (46). In AD, the cutaneous inflammatory lesions are characterized by a type 2 inflammatory milieu including M2-polarized macrophages infiltrating the diseased skin (20, 47, 48). A shift to the M1 phenotype of macrophages would reduce *S. aureus* colonization and infections in AD patients. However, this study illustrates that M1-polarized MDMs of AD patients express an increased amount of CYLD and have an impaired capacity to kill *S. aureus*. The functional importance of CYLD is shown in THP-1 macrophages, which only incompletely shift to an M1 phenotype as illustrated by reduced CD80 expression and TNF production and by the improved control of *S. aureus* in *CYLD*-deficient M1-polarized MDMs of healthy blood donors and THP-1 cells.

The present study newly identifies that CYLD binds to STAT1 upon *S. aureus* infection of M1-polarized human MDMs. This interaction resulted in a CYLD-dependent reduction of K63 polyubiquitination of STAT1 and an impaired STAT1 phosphorylation. CYLD inhibited both the phosphorylation at STAT1 (Y701), which is important for nuclear accumulation of the STAT1 transcription factor, and the subsequent nuclear phosphorylation at STAT1 (S727), which is required for gene transcription (49, 50). The functional importance of STAT1 for the control of *S. aureus* was illustrated by the impaired control of *S. aureus* in M1-polarized MDMs upon siRNA-mediated inhibition of STAT1.

The induction of an anti-staphylococcal function of macrophages also requires the engagement of pathogen pattern recognition receptors leading to the activation of NF-κB (12). In the present study, the pre-stimulation with the TLR4 agonist LPS and the subsequent *S. aureus* infection activating TLR2 and NOD2 by lipoteichoic acid and muramyl dipeptide led to the activation of NF-κB and NF-κB-dependent production of anti-bacterial ROS. Production of ROS is important and has a superior role in comparison to NO in controlling *S. aureus* infections (13, 51–55). In agreement with previous studies, we detected an interaction of CYLD with RIPK2, TRAF6, and IKKγ/NEMO, which are critical signaling molecules in the NOD2 and TLR2/4 pathways leading to NF-κB activation (29, 30, 32, 38). CYLD inhibits the activation of these signaling molecules by its K63 deubiquitinating activity. The key role of CYLD-mediated NF-κB inhibition as a critical factor impairing control of *S. aureus* in macrophages is proven by the strong increase and abolishment of differences in CFUs between CYLD-competent and CYLD-deficient macrophages. Since the numbers of *S. aureus* were identical in both NF-κB-inhibited and STAT1 siRNA-treated WT and *CYLD* ^−/−^ macrophages, the two pathways determine independently from each other the control of *S. aureus* and cannot compensate for each other.

Of note, infection with *S. aureus* led to a CYLD-independent and equal activation of MAP kinases including c-Jun-N-terminal kinase (JNK), p38, and ERK in CYLD-competent and CYLD-deficient MDMs. Previously, we have shown that the inhibition of RIPK2 by CYLD impairs the activation of MAP kinases and ERK1/2-induced autophagy in *Listeria monocytogenes*-infected macrophages (38). In the present study, MAP kinases, in particular JNK, were equally activated in CYLD-deficient and CYLD-competent MDMs. This difference may be explained by the manipulation of MAP kinases by *S. aureus*, which may undermine JNK function by a yet unresolved mechanism to induce its persistence in macrophages (56). The pathogen *S. aureus* has been found to prominently colonize and thereby overgrow commensal microbiota on the skin of AD patients, aggravating AD on different levels. Its role in the disease is further underlined by the finding that coagulase-negative staphylococci, which are able to suppress the growth of *S. aureus*, were less present on the skin of babies who had developed AD at the second study visit after 1 year (57). Healthy mice treated with *S. aureus* strains derived from AD patients develop a robust immune reaction including type 2 cytokines and induction of skin-resident T cells (58). While keratinocytes act as sensors of *S. aureus* infection (59), *S. aureus* is capable of infiltrating keratinocytes and immune cells (58, 60), thereby reducing the effectivity of antimicrobial or antibiotic treatment. Immune cells, which are crucial for the final clearance, are efficiently repressed by evasion strategies (17). Strengthening the hosts’ cellular mechanism to control *S. aureus* infection could therefore be a potent therapy option in AD.

Our data show that M1 polarization of macrophages augments the anti-staphylococcal activity of human and murine macrophages. Unleashing the CYLD break on NF-κB further enhances the control of *S. aureus* of M1-polarized macrophages. The *in vivo* data illustrating that *Cyld* ^−/−^ mice have an improved course and control of *S. aureus* in acute and chronic systemic infection further corroborate that CYLD is a potential therapeutic target to reduce *S. aureus* colonization and infection.

### The potential of CYLD inhibition in infectious diseases

CYLD is known to negatively regulate various signaling pathways, including NF-κB (61, 62), MAPK (38), and JAK/STAT (37, 38, 63) pathways, which are crucial for the inflammatory response. Our previous studies have identified CYLD as an inhibitor of immune responses during bacterial (37, 38) and parasitic infections (64), and inhibition of CYLD resulted in a positive disease outcome. Inhibition of CYLD in hepatocytes promoted IL-6/STAT3-mediated fibrin production and prevented the spread of *L. monocytogenes* (37). Ablation of CYLD in macrophages enhanced NF-κB-mediated production of reactive oxygen species, resulting in improved clearance of *L. monocytogenes* (38). Depletion of CYLD in T cells attenuates T-cell responses, prevents blood–brain barrier disruption, and protects mice from experimental cerebral malaria (64). Furthermore, Lim et al. (2007) showed that CYLD deficiency protected mice from *S. pneumoniae* pneumolysin (PLY)-induced Acute lung injury (ALI) and lethality (34). In the current study, we identified CYLD as an inhibitor of the anti-*S. aureus* immune response in human and murine macrophages. These studies highlight the potential of CYLD as a therapeutic target in infectious diseases. However, it should be noted that CYLD negatively regulates NF-κB-dependent inflammation during non-typeable *H. influenzae* infection and protects mice from deleterious inflammatory responses (65). Similarly, CYLD impairs inflammation and protects mice from *E. coli*-induced pneumonia (35), suggesting that the protective or detrimental function of CYLD in infectious diseases depends on the underlying infection.

### Impact on macrophage polarization

M1 macrophages are crucial in defending against pathogens through phagocytosis and the secretion of pro-inflammatory cytokines. Several bacteria, including *S. aureus* (66), *Mycobacterium* (67), *Salmonella* (68), and *Coxiella* (69), promote M2 polarization to evade the pro-inflammatory response and enhance their survival within macrophages. Ablation of CYLD would foster M1 polarization, enhancing their antimicrobial activities and leading to improved outcomes in these bacterial infections.

### Therapeutic strategies

Commercially available small molecule inhibitors that target the USP family of deubiquitinases, such as NSC-687852, NSC112200, and WP1130, could be used to inhibit CYLD. More specifically, subquinocin, a small molecule inhibitor primarily targeting CYLD, could serve as a potential therapeutic strategy against infectious diseases (70). Additionally, proteolysis-targeting chimeras (PROTACs), which utilize the ubiquitin–proteasome system to target specific proteins for degradation (71), could be used to specifically target CYLD for proteasomal degradation, making it a promising therapeutic option.

## Data Availability

The datasets presented in this study can be found in online repositories. The names of the repository/repositories and accession number(s) can be found below: EGAD00001010106 (EGA; https://ega-archive.org).

## Supplementary material

Supplementary Figure 1 Increased CYLD protein expression in the epidermal skin of atopic dermatitis patients. Exemplary pictures of CYLD protein expression (brown, AEC) in the skin of three patients with AD and one healthy control. Sections were immunostained with a polyclonal rabbit anti-CYLD antibody or a respective isotype control and counterstained with hemalum. Scale bar = 100µm.

## References

[1] Edslev SM Olesen CM Norreslet LB Ingham AC Iversen S Lilje B. Staphylococcal communities on skin are associated with atopic dermatitis and disease severity. Microorganisms. (2021) 9. doi: 10.3390/microorganisms9020432 PMC7921937 33669791

[2] Guzik TJ Bzowska M Kasprowicz A Czerniawska-Mysik G Wojcik K Szmyd D. Persistent skin colonization with Staphylococcus aureus in atopic dermatitis: relationship to clinical and immunological parameters. Clin Exp Allergy. (2005) 35:448–55. doi: 10.1111/j.1365-2222.2005.02210.x 15836752

[3] Kong HH Oh J Deming C Conlan S Grice EA Beatson MA. Temporal shifts in the skin microbiome associated with disease flares and treatment in children with atopic dermatitis. Genome Res. (2012) 22:850–9. doi: 10.1101/gr.131029.111 PMC3337431 22310478

[4] Saheb Kashaf S Harkins CP Deming C Joglekar P Conlan S Holmes CJ. Staphylococcal diversity in atopic dermatitis from an individual to a global scale. Cell Host Microbe. (2023) 31:578–592 e576. doi: 10.1016/j.chom.2023.03.010 37054678 PMC10151067

[5] Wichmann K Uter W Weiss J Breuer K Heratizadeh A Mai U. Isolation of alpha-toxin-producing Staphylococcus aureus from the skin of highly sensitized adult patients with severe atopic dermatitis. Br J Dermatol. (2009) 161:300–5. doi: 10.1111/j.1365-2133.2009.09229.x 19438853

[6] Deng L Costa F Blake KJ Choi S Chandrabalan A Yousuf MS. S. aureus drives itch and scratch-induced skin damage through a V8 protease-PAR1 axis. Cell. (2023) 186:5375–5393 e5325. doi: 10.1016/j.cell.2023.10.019 37995657 PMC10669764

[7] Geoghegan JA Irvine AD Foster TJ. Staphylococcus aureus and atopic dermatitis: A complex and evolving relationship. Trends Microbiol. (2018) 26:484–97. doi: 10.1016/j.tim.2017.11.008 29233606

[8] Langer K Breuer K Kapp A Werfel T. Staphylococcus aureus-derived enterotoxins enhance house dust mite-induced patch test reactions in atopic dermatitis. Exp Dermatol. (2007) 16:124–9. doi: 10.1111/j.1600-0625.2006.00523.x 17222226

[9] Breuer K S HA Kapp A Werfel T. Staphylococcus aureus: colonizing features and influence of an antibacterial treatment in adults with atopic dermatitis. Br J Dermatol. (2002) 147:55–61. doi: 10.1046/j.1365-2133.2002.04872.x 12100185

[10] Spaan AN Surewaard BG Nijland R van Strijp JA. Neutrophils versus Staphylococcus aureus: a biological tug of war. Annu Rev Microbiol. (2013) 67:629–50. doi: 10.1146/annurev-micro-092412-155746 23834243

[11] von Kockritz-Blickwede M Winstel V. Molecular prerequisites for neutrophil extracellular trap formation and evasion mechanisms of staphylococcus aureus. Front Immunol. (2022) 13:836278. doi: 10.3389/fimmu.2022.836278 35237275 PMC8884242

[12] Pidwill GR Gibson JF Cole J Renshaw SA Foster SJ. The role of macrophages in staphylococcus aureus infection. Front Immunol. (2020) 11:620339. doi: 10.3389/fimmu.2020.620339 33542723 PMC7850989

[13] Surewaard BG Deniset JF Zemp FJ Amrein M Otto M Conly J. Identification and treatment of the Staphylococcus aureus reservoir in *vivo* . J Exp Med. (2016) 213:1141–51. doi: 10.1084/jem.20160334 PMC4925027 27325887

[14] Chan LC Rossetti M Miller LS Filler SG Johnson CW Lee HK. Protective immunity in recurrent Staphylococcus aureus infection reflects localized immune signatures and macrophage-conferred memory. Proc Natl Acad Sci U.S.A. (2018) 115:E11111–9. doi: 10.1073/pnas.1808353115 PMC6255181 30297395

[15] Gresham HD Lowrance JH Caver TE Wilson BS Cheung AL Lindberg FP. Survival of Staphylococcus aureus inside neutrophils contributes to infection. J Immunol. (2000) 164:3713–22. doi: 10.4049/jimmunol.164.7.3713 10725730

[16] Soong G Paulino F Wachtel S Parker D Wickersham M Zhang D. Methicillin-resistant Staphylococcus aureus adaptation to human keratinocytes. mBio. (2015) 6. doi: 10.1128/mBio.00289-15 PMC4453558 25900653

[17] Thammavongsa V Kim HK Missiakas D Schneewind O. Staphylococcal manipulation of host immune responses. Nat Rev Microbiol. (2015) 13:529–43. doi: 10.1038/nrmicro3521 PMC4625792 26272408

[18] Tuchscherr L Medina E Hussain M Volker W Heitmann V Niemann S. Staphylococcus aureus phenotype switching: an effective bacterial strategy to escape host immune response and establish a chronic infection. EMBO Mol Med. (2011) 3:129–41. doi: 10.1002/emmm.201000115 PMC3395110 21268281

[19] Beck LA Cork MJ Amagai M De Benedetto A Kabashima K Hamilton JD. Type 2 inflammation contributes to skin barrier dysfunction in atopic dermatitis. JID Innov. (2022) 2:100131. doi: 10.1016/j.xjidi.2022.100131 36059592 PMC9428921

[20] Zhang B Roesner LM Traidl S Koeken V Xu CJ Werfel T. Single-cell profiles reveal distinctive immune response in atopic dermatitis in contrast to psoriasis. Allergy. (2023) 78:439–53. doi: 10.1111/all.15486 35986602

[21] Brunner PM Silverberg JI Guttman-Yassky E Paller AS Kabashima K Amagai M. Increasing comorbidities suggest that atopic dermatitis is a systemic disorder. J Invest Dermatol. (2017) 137:18–25. doi: 10.1016/j.jid.2016.08.022 27771048

[22] Droitcourt C Vittrup I Kerbrat S Egeberg A Thyssen JP. Risk of systemic infections in adults with atopic dermatitis: A nationwide cohort study. J Am Acad Dermatol. (2021) 84:290–9. doi: 10.1016/j.jaad.2020.07.111 32750384

[23] Jin SP Lee K Bang YJ Jeon YH Jung S Choi SJ. Mapping the immune cell landscape of severe atopic dermatitis by single-cell RNA-seq. Allergy. (2024) 79:1584–97. doi: 10.1111/all.16121 38817208

[24] Chau V Tobias JW Bachmair A Marriott D Ecker DJ Gonda DK. A multiubiquitin chain is confined to specific lysine in a targeted short-lived protein. Science. (1989) 243:1576–83. doi: 10.1126/science.2538923 2538923

[25] Meyer HJ Rape M. Enhanced protein degradation by branched ubiquitin chains. Cell. (2014) 157:910–21. doi: 10.1016/j.cell.2014.03.037 PMC4028144 24813613

[26] Rahman S Wolberger C. Breaking the K48-chain: linking ubiquitin beyond protein degradation. Nat Struct Mol Biol. (2024) 31:216–8. doi: 10.1038/s41594-024-01221-w PMC11730971 38366227

[27] Komander D Reyes-Turcu F Licchesi JD Odenwaelder P Wilkinson KD Barford D. Molecular discrimination of structurally equivalent Lys 63-linked and linear polyubiquitin chains. EMBO Rep. (2009) 10:466–73. doi: 10.1038/embor.2009.55 PMC2680876 19373254

[28] Marin-Rubio JL Raote I Inns J Dobson-Stone C Rajan N. CYLD in health and disease. Dis Model Mech. (2023) 16. doi: 10.1242/dmm.050093 PMC10320722 37387450

[29] Brummelkamp TR Nijman SM Dirac AM Bernards R. Loss of the cylindromatosis tumour suppressor inhibits apoptosis by activating NF-kappaB. Nature. (2003) 424:797–801. doi: 10.1038/nature01811 12917690

[30] Kovalenko A Chable-Bessia C Cantarella G Israel A Wallach D Courtois G. The tumour suppressor CYLD negatively regulates NF-kappaB signalling by deubiquitination. Nature. (2003) 424:801–5. doi: 10.1038/nature01802 12917691

[31] Reiley WW Jin W Lee AJ Wright A Wu X Tewalt EF. Deubiquitinating enzyme CYLD negatively regulates the ubiquitin-dependent kinase Tak1 and prevents abnormal T cell responses. J Exp Med. (2007) 204:1475–85. doi: 10.1084/jem.20062694 PMC2118606 17548520

[32] Trompouki E Hatzivassiliou E Tsichritzis T Farmer H Ashworth A Mosialos G. CYLD is a deubiquitinating enzyme that negatively regulates NF-kappaB activation by TNFR family members. Nature. (2003) 424:793–6. doi: 10.1038/nature01803 12917689

[33] Lim JH Jono H Koga T Woo CH Ishinaga H Bourne P. Tumor suppressor CYLD acts as a negative regulator for non-typeable Haemophilus influenza-induced inflammation in the middle ear and lung of mice. PloS One. (2007) 2:e1032. doi: 10.1371/journal.pone.0001032 17925880 PMC2001183

[34] Lim JH Stirling B Derry J Koga T Jono H Woo CH. Tumor suppressor CYLD regulates acute lung injury in lethal Streptococcus pneumoniae infections. Immunity. (2007) 27:349–60. doi: 10.1016/j.immuni.2007.07.011 17723219

[35] Lim JH Ha UH Woo CH Xu H Li JD. CYLD is a crucial negative regulator of innate immune response in Escherichia coli pneumonia. Cell Microbiol. (2008) 10:2247–56. doi: 10.1111/j.1462-5822.2008.01204.x 18643924

[36] Wurm R Just S Wang X Wex K Schmid U Blanchard N. Protective dendritic cell responses against listeriosis induced by the short form of the deubiquitinating enzyme CYLD are inhibited by full-length CYLD. Eur J Immunol. (2015) 45:1366–76. doi: 10.1002/eji.201445116 25675948

[37] Nishanth G Deckert M Wex K Massoumi R Schweitzer K Naumann M. CYLD enhances severe listeriosis by impairing IL-6/STAT3-dependent fibrin production. PloS Pathog. (2013) 9:e1003455. doi: 10.1371/journal.ppat.1003455 23825949 PMC3695090

[38] Wex K Schmid U Just S Wang X Wurm R Naumann M. Receptor-interacting protein kinase-2 inhibition by CYLD impairs antibacterial immune responses in macrophages. Front Immunol. (2015) 6:650. doi: 10.3389/fimmu.2015.00650 26834734 PMC4717182

[39] Nagy N Dubois A Szell M Rajan N. Genetic testing in CYLD cutaneous syndrome: an update. Appl Clin Genet. (2021) 14:427–44. doi: 10.2147/TACG.S288274 PMC8566010 34744449

[40] Ji YX Huang Z Yang X Wang X Zhao LP Wang PX. The deubiquitinating enzyme cylindromatosis mitigates nonalcoholic steatohepatitis. Nat Med. (2018) 24:213–23. doi: 10.1038/nm.4461 29291351

[41] Zhou JJ Li H Li L Li Y Wang PH Meng XM. CYLD mediates human pulmonary artery smooth muscle cell dysfunction in congenital heart disease-associated pulmonary arterial hypertension. J Cell Physiol. (2021) 236:6297–311. doi: 10.1002/jcp.30298 33507567

[42] Dobson-Stone C Hallupp M Shahheydari H Ragagnin AMG Chatterton Z Carew-Jones F. CYLD is a causative gene for frontotemporal dementia - amyotrophic lateral sclerosis. Brain. (2020) 143:783–99. doi: 10.1093/brain/awaa039 PMC7089666 32185393

[43] Uhlen M Oksvold P Fagerberg L Lundberg E Jonasson K Forsberg M. Towards a knowledge-based human protein atlas. Nat Biotechnol. (2010) 28:1248–50. doi: 10.1038/nbt1210-1248 21139605

[44] Massoumi R Chmielarska K Hennecke K Pfeifer A Fassler R. Cyld inhibits tumor cell proliferation by blocking Bcl-3-dependent NF-kappaB signaling. Cell. (2006) 125:665–77. doi: 10.1016/j.cell.2006.03.041 16713561

[45] Yajjala VK Thomas VC Bauer C Scherr TD Fischer KJ Fey PD. Resistance to acute macrophage killing promotes airway fitness of prevalent community-acquired staphylococcus aureus strains. J Immunol. (2016) 196:4196–203. doi: 10.4049/jimmunol.1600081 PMC4868659 27053759

[46] Asai A Tsuda Y Kobayashi M Hanafusa T Herndon DN Suzuki F. Pathogenic role of macrophages in intradermal infection of methicillin-resistant Staphylococcus aureus in thermally injured mice. Infect Immun. (2010) 78:4311–9. doi: 10.1128/IAI.00642-10 PMC2950336 20679444

[47] Hashimoto T Yokozeki H Karasuyama H Satoh T. IL-31-generating network in atopic dermatitis comprising macrophages, basophils, thymic stromal lymphopoietin, and periostin. J Allergy Clin Immunol. (2023) 151:737–746 e736. doi: 10.1016/j.jaci.2022.11.009 36410530

[48] Kasraie S Werfel T. Role of macrophages in the pathogenesis of atopic dermatitis. Mediators Inflammation. (2013), 942375. doi: 10.1155/2013/942375 PMC3603294 23533313

[49] Sadzak I Schiff M Gattermeier I Glinitzer R Sauer I Saalmuller A. Recruitment of Stat1 to chromatin is required for interferon-induced serine phosphorylation of Stat1 transactivation domain. Proc Natl Acad Sci U.S.A. (2008) 105:8944–9. doi: 10.1073/pnas.0801794105 PMC2435588 18574148

[50] Varinou L Ramsauer K Karaghiosoff M Kolbe T Pfeffer K Muller M. Phosphorylation of the Stat1 transactivation domain is required for full-fledged IFN-gamma-dependent innate immunity. Immunity. (2003) 19:793–802. doi: 10.1016/s1074-7613(03)00322-4 14670297

[51] Hashimoto M Tawaratsumida K Kariya H Aoyama K Tamura T Suda Y. Lipoprotein is a predominant Toll-like receptor 2 ligand in Staphylococcus aureus cell wall components. Int Immunol. (2006) 18:355–62. doi: 10.1093/intimm/dxh374 16373361

[52] Pizzolla A Hultqvist M Nilson B Grimm MJ Eneljung T Jonsson IM. Reactive oxygen species produced by the NADPH oxidase 2 complex in monocytes protect mice from bacterial infections. J Immunol. (2012) 188:5003–11. doi: 10.4049/jimmunol.1103430 PMC3566649 22491245

[53] Schaffler H Demircioglu DD Kuhner D Menz S Bender A Autenrieth IB. NOD2 stimulation by Staphylococcus aureus-derived peptidoglycan is boosted by Toll-like receptor 2 costimulation with lipoproteins in dendritic cells. Infect Immun. (2014) 82:4681–8. doi: 10.1128/IAI.02043-14 PMC4249339 25156723

[54] Takeuchi O Hoshino K Kawai T Sanjo H Takada H Ogawa T. Differential roles of TLR2 and TLR4 in recognition of gram-negative and gram-positive bacterial cell wall components. Immunity. (1999) 11:443–51. doi: 10.1016/s1074-7613(00)80119-3 10549626

[55] Takeuchi O Takeda K Hoshino K Adachi O Ogawa T Akira S. Cellular responses to bacterial cell wall components are mediated through MyD88-dependent signaling cascades. Int Immunol. (2000) 12:113–7. doi: 10.1093/intimm/12.1.113 10607756

[56] Watanabe I Ichiki M Shiratsuchi A Nakanishi Y. TLR2-mediated survival of Staphylococcus aureus in macrophages: a novel bacterial strategy against host innate immunity. J Immunol. (2007) 178:4917–25. doi: 10.4049/jimmunol.178.8.4917 17404273

[57] Kennedy EA Connolly J Hourihane JO Fallon PG McLean WHI Murray D. Skin microbiome before development of atopic dermatitis: Early colonization with commensal staphylococci at 2 months is associated with a lower risk of atopic dermatitis at 1 year. J Allergy Clin Immunol. (2017) 139:166–72. doi: 10.1016/j.jaci.2016.07.029 PMC5207796 27609659

[58] Braun C Badiou C Guironnet-Paquet A Iwata M Lenief V Mosnier A. Staphylococcus aureus-specific skin resident memory T cells protect against bacteria colonization but exacerbate atopic dermatitis-like flares in mice. J Allergy Clin Immunol. (2024) 154:355–74. doi: 10.1016/j.jaci.2024.03.032 38734386

[59] Bitschar K Wolz C Krismer B Peschel A Schittek B. Keratinocytes as sensors and central players in the immune defense against Staphylococcus aureus in the skin. J Dermatol Sci. (2017) 87:215–20. doi: 10.1016/j.jdermsci.2017.06.003 28655473

[60] Kintarak S Whawell SA Speight PM Packer S Nair SP. Internalization of Staphylococcus aureus by human keratinocytes. Infect Immun. (2004) 72:5668–75. doi: 10.1128/IAI.72.10.5668-5675.2004 PMC517534 15385465

[61] Trompouki E Hatzivassiliou E Tsichritzis T Farmer H Ashworth A Mosialos G. CYLD is a deubiquitinating enzyme that negatively regulates NF-κB activation by TNFR family members. Nature. (2003) 424:793–6. doi: 10.1038/nature01803 12917689

[62] Jono H Lim JH Chen LF Xu H Trompouki E Pan ZK. NF-kappaB is essential for induction of CYLD, the negative regulator of NF-kappaB: evidence for a novel inducible autoregulatory feedback pathway. J Biol Chem. (2004) 279:36171–4. doi: 10.1074/jbc.M406638200 15226292

[63] Huyen NT Ngoc NT Giang NH Trang DT Hanh HH Binh VD. CYLD stimulates macrophage phagocytosis of leukemic cells through STAT1 signalling in acute myeloid leukemia. PloS One. (2023) 18:e0283586. doi: 10.1371/journal.pone.0283586 37549179 PMC10406188

[64] Schmid U Stenzel W Koschel J Raptaki M Wang X Naumann M. The deubiquitinating enzyme cylindromatosis dampens CD8(+) T cell responses and is a critical factor for experimental cerebral malaria and blood-brain barrier damage. Front Immunol. (2017) 8:27. doi: 10.3389/fimmu.2017.00027 28203236 PMC5285367

[65] Wang WY Komatsu K Huang Y Wu J Zhang W Lee JY. CYLD negatively regulates nontypeable Haemophilus influenzae-induced IL-8 expression via phosphatase MKP-1-dependent inhibition of ERK. PloS One. (2014) 9:e112516. doi: 10.1371/journal.pone.0112516 25389768 PMC4229244

[66] Xu F Kang Y Zhang H Piao Z Yin H Diao R. Akt1-mediated regulation of macrophage polarization in a murine model of Staphylococcus aureus pulmonary infection. J Infect Dis. (2013) 208:528–38. doi: 10.1093/infdis/jit177 23613163

[67] Sha S Shi Y Tang Y Jia L Han X Liu Y. Mycobacterium tuberculosis Rv1987 protein induces M2 polarization of macrophages through activating the PI3K/Akt1/mTOR signaling pathway. Immunol Cell Biol. (2021) 99:570–85. doi: 10.1111/imcb.12436 33469941

[68] Leiba J Sipka T Begon-Pescia C Bernardello M Tairi S Bossi L. Dynamics of macrophage polarization support Salmonella persistence in a whole living organism. Elife. (2024) 13. doi: 10.7554/eLife.89828 PMC10830131 38224094

[69] Benoit M Barbarat B Bernard A Olive D Mege JL. Coxiella burnetii, the agent of Q fever, stimulates an atypical M2 activation program in human macrophages. Eur J Immunol. (2008) 38:1065–70. doi: 10.1002/eji.200738067 18350541

[70] Yamanaka S Sato Y Oikawa D Goto E Fukai S Tokunaga F. Subquinocin, a small molecule inhibitor of CYLD and USP-family deubiquitinating enzymes, promotes NF-kappaB signaling. Biochem Biophys Res Commun. (2020) 524:1–7. doi: 10.1016/j.bbrc.2019.12.049 31898971

[71] Neklesa TK Winkler JD Crews CM. Targeted protein degradation by PROTACs. Pharmacol Ther. (2017) 174:138–44. doi: 10.1016/j.pharmthera.2017.02.027 28223226

