# Supplementary figures and images for: Ablation of the deubiquitinating enzyme cylindromatosis (CYLD) augments STAT1-mediated M1 macrophage polarization and fosters Staphylococcus aureus control
Source: Front Immunol. 2025 Jan 28;16:1507989. doi: 10.3389/fimmu.2025.1507989 (PMC11827430; doi:10.3389/fimmu.2025.1507989)

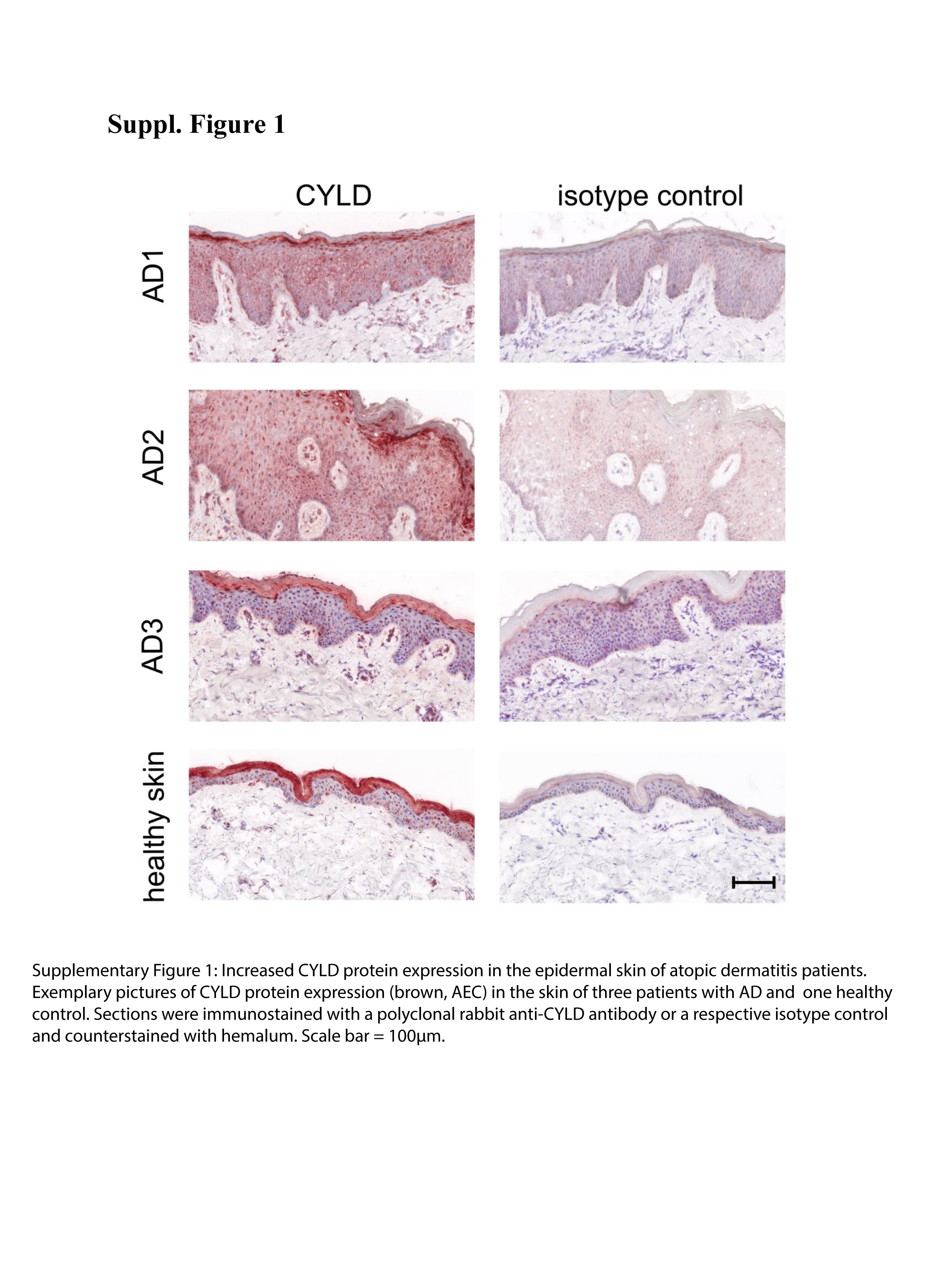

Supplement: Supplementary Figure 1 — Increased CYLD protein expression in the epidermal skin of atopic dermatitis patients. Exemplary pictures of CYLD protein expression (brown, AEC) in the skin of three patients with AD and one healthy control. Sections were immunostained with a polyclonal rabbit anti-CYLD antibody or a respective isotype control and counterstained with hemalum. Scale bar = 100µm. [file Image1.tif]
